# Effect of Channel Blockers on the Smooth Muscle of the Adult Crop of the Queen Blowfly, *Phormia regina*

**DOI:** 10.1673/031.013.9701

**Published:** 2013-09-30

**Authors:** John G. Stoffolano, Laura Danai, James Chambers

**Affiliations:** 1 Department of Plant, Soil, and Insect Sciences, University of Massachusetts, Amherst, MA 01003, USA; 2 Department of Chemistry, University of Massachusetts, Amherst, MA 01003, USA

**Keywords:** 9-AC, amiloride, benzyltrimethylammonium chloride, CIC channels, foregut, visceral muscle

## Abstract

Few studies have examined the various factors affecting the rate of contraction of the supercontractile muscles of the crop lobes of adult *Phormia regina* Meigen (Diptera: Calliphoridae). Using an *in situ* bioassay of the crop organ, various ion channel blockers were tested and it was demonstrated that in all cases the blockers (i.e., against the following conductances: Cl^-^ , Ca^2+^ , Na^+^, and a FMRF-amide action) significantly reduced the contraction rates of the crop lobes, which were filled with 4.5 µL of 1.0 M sucrose containing 10 mM of the dye amaranth. Benzyltrimethylammonium chloride, never before reported for its effect on insect muscle, was as effective in suppressing crop muscle contraction as benzethonium chloride, which is a reported agonist of dromyosuppressin.

## Introduction

*Phormia regina* Meigen (Diptera: Calliphoridae) is an important model in feeding behavior studies because the mechanisms regulating food ingestion are the best understood of all insect species (Dethier 1976). Adult *Phormia* possess a diverticulated crop organ used for the storage of protein and carbohydrates before these nutrients make their way into the midgut. The rate at which the muscles of this organ contract have an important physiological function in the fly, and it is believed the contraction rate determines when food passes out of the crop lobes into the midgut, or out of the mouth (i.e., as a droplet or regurgitant), and at what rate. Thomson (1975a), commenting on Gelperin's (1966) paper, suggested that because crop emptying is independent of both nervous and endocrine direct control, it is likely based on a myogenic mechanism. Consequently, the crop muscles should become the main focus of study in order to understand both crop emptying and filling. It has been reported the contents and volume of the crop lobes affect the rate of contraction, but little has been done to determine what other factors may influence these muscles. Recently, it was shown, using both an *in vitro* bioassay and electrophysiological recordings, that exogenous serotonin added to the crop organ system significantly increased pump 4 (P4) contractions, while removing calcium from the system significantly decreased muscular contractions (Liscia et al. 2012). The crop organ is composed of two major regions, the crop duct and the crop lobes. Each of these regions contains a series of pumps (P) and sphincters, as initially illustrated by Thomson (1975b) and later modified by Liscia et al. (2012). Recordings from the crop lobe muscles in response to serotonin mirrored the results for pump 4 (unpublished data). However, contraction of crop lobe muscles (P5) and pump 4 (P4) are not always equivalent and may be under different controls (Stoffolano et al. 2010). Because few recent studies have focused on this neglected part of the digestive system, the control of fore-gut contraction is still poorly understood (Miller 1975). Our study focused on the types of ion channels that are present in these muscles and if their contraction can be blocked using specific channel blockers.

## Materials and Methods

### Maintaining flies

Flies were obtained from a colony maintained as previously described by Stoffolano (1974) and kept at 27° C, 30% RH, and a photoperiod of 16:8 L:D. Eggs were collected and placed in 473 mL plastic cups containing an artificial diet (Stoffolano et al. 2010). After several days of growth, the cup was placed in a container with sand, and when the larvae were ready to pupate, they crawled into the sand. Pupae were obtained and transferred to another 473 mL plastic cup, which was then placed into a metal cage (20.3 cm on each side). When the adult flies emerged, they were fed a 0.126 M sucrose solution.

### Testing crop lobes for contraction rates

Tests were performed during the fourth day, post-emergence. A starvation period of 16 hr on the third day, prior to testing, provided flies on day 4 with empty crops. Females were removed from the cages and placed in 1 mL vials capped with Parafilm® with holes for ventilation. All flies were allowed to habituate to their vials for 1 hr. After habituation, a 4.5 µL solution of 1.0 M sucrose containing 10 mM of the dye amaranth (Sigma-Aldrich, www.sigmaaldrich.com) was added to each vial. Amaranth was added to the sucrose solution because Thomson (1975b) reported it had no effect on taste but made it easier to visualize the lobes during dissection/counting. In all of the experiments, individual flies were allowed 2–4 min to consume the solution and then placed in the freezer for cold immobilization for 5 min. Once immobilized, each fly was pinned down, ventral side up, on a wax plate containing 20 mL of *Phormia* saline (Chen and Friedman 1975). The abdomen was opened, starting from the terminal end of the fly to the thorax, exposing the lobes (P5). Using a dissecting microscope, counts were taken on P5 after 1 min of acclimatization of the preparation to the saline in all experiments. In order to determine the crop lobe contraction rate, a point was chosen on the crop lobes, and every time the muscles at that point contracted it was recorded as one contraction. Furthermore, a similar point where contraction was evident was chosen for all the crops tested in order to maintain consistency.

**Figure 1.**
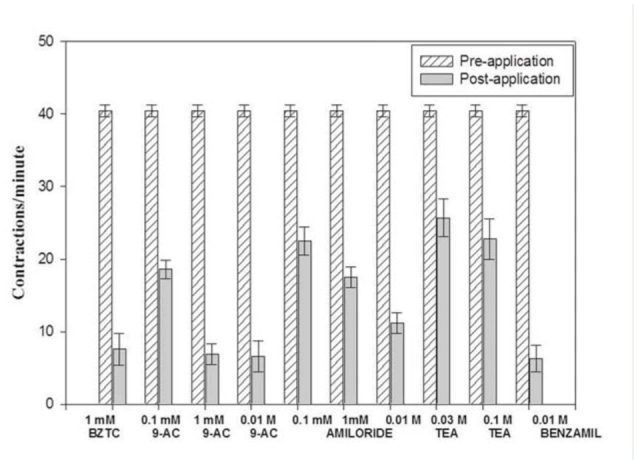
Effect of three different chemicals, using the handheld profusion technique with an *in situ* bioassay preparation, on the rates of crop lobe muscle (P5) contractions for adult, female *Phormia regina*. All crops were tested on the fourth day of post-emergence after imbibing 4.5 µL of 1.0 M sucrose. 9-AC = 9-anthracenecarboxylic acid. High quality figures are available online.

**Figure 2.**
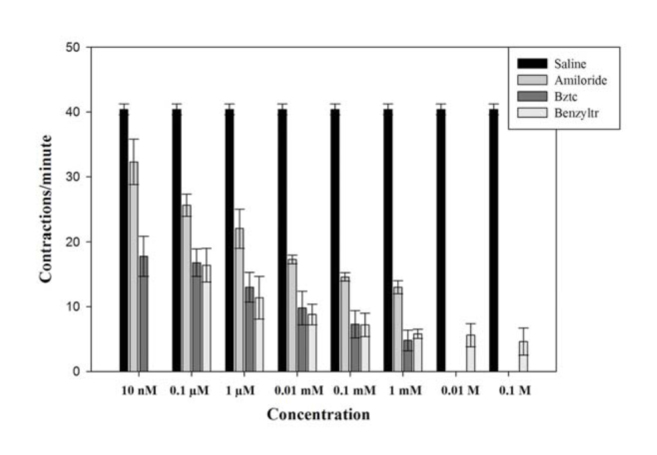
Effect of amiloride, Bztc, and benzyltrimethylammonium (Benzyltr) chloride on crop lobe muscle (P5) contraction rates (± SE) in female *Phormia regina* tested on day 4 post-eclosion after imbibing 4.5 µL of 1.0 M sucrose using the gravity perfusion system and expressed as an IC_50_ value. High quality figures are available online.

Unless stated otherwise, 25 µL of the desired chemical solution was placed directly on top of the contracting crop lobes (the tip of the pipette never touched the preparation) and contractions were recorded for 1 extra minute.

### Testing the effects of various ion channel blockers on crop lobe muscle contraction

Initially, experiments were performed using a hand, two pipette transfer system (results in Figure 1). The hand transfer system involved sucking off the solution, bathing the preparation with one hand using a pipette, while at the same time adding with the other hand and pipette the new solution. The same overall procedures were performed throughout the experiments as stated above, but when measuring the 50% inhibitory concentration (IC_50_), each fly was exposed to a progressively higher concentration (making sure to wash the chemical with saline for 30 sec in between concentrations). This technique was facilitated by using an ALA Science VC3-8C (www.alascience.com) 94 gravity-fed perfusion system (results in Figure 2). Chemicals were also tested at lower concentrations in order to calculate their IC_50_. IC_50_ is a measure of the effectiveness of a compound in inhibiting biological or biochemical functions. In other words, IC_50_ indicates how much of a chemical is needed to inhibit a biological process by half. All test chemicals were obtained from Sigma-Aldrich.

### Statistics

A t-test (PROC TTEST, SAS Institute 2009) was used to determine the effect of different chemicals on crop lobe (P5) contraction by comparing the contraction rate after application to the basal contraction rate (in the absence of chemicals).

## Results

### Effect of chemicals on crop lobe muscle contraction rates using a hand-operated perfusion technique

The average basal rate of crop lobe muscle (P5) contractions for all flies tested was 40.37 (SE ± 0.82) contractions/min. Benzethonium chloride (Bztc), previously reported to be an agonist of dromyosuppressin (Richer et al. 2000), was initially tested at only one concentration (0.001 M) as a check on the methodology previously reported from our laboratory. Contraction rates decreased to 7.6 ± 2.2 after application of the solution and were significantly different from the controls (*p* ≤ 0.0001) (Figure 1). Bztc was also tested at various concentrations using the gravity perfusion system, with results presented in Figure 2.

All three concentrations of 9-anthracenecarboxylic acid (9-AC) produced highly significant (*p* ≤ 0.0001) decreases in contraction rates when compared to the controls (Figure 1). All amiloride solutions showed a highly significant effect (*p* ≤ .0001) and, in a dose-dependent manner, caused a decrease in muscle contractions (Figure 1). Results for amiloride at varying concentrations to determine the IC_50_ value are presented in Figure 2.

### Effect of chemicals on crop lobe muscle contraction rates using the gravity-fed perfusion system

In order to determine the IC_50_ values, three chemicals were selected for study: benzethonium chloride, benzyltrimethylammonium chloride, and amiloride. Results for Bztc showed a decrease in the contraction rates from the controls, and its effects were in a dose-dependent manner, with an IC_50_ value of 10 nM (N = 6) (Figure 2). Benzyltrimethylammonium chloride and amiloride produced similar dose-dependent responses with IC_50_ values of 10 nM (N = 5) and 0.01 mM (N = 3), respectively (Figure 2).

## Discussion

Few studies have investigated the various factors regulating the contraction of any of the supercontractile muscles of the adult dipteran crop. Knight (1962) appears to be the first, followed by Gelperin (1966) and Thomson (1975a, b), to examine the movement of fluids in the adult crop of *P. regina*. It wasn't until Nichols (1992) and Richer et al. (2000) showed the neuropeptide dromyosuppressin dramatically reduced muscle contractions of the adult crop lobes in *Drosophila melanogaster* and *P. regina*, respectively, that investigators started to look at what other factors might affect muscle contractions of this organ system.

### Channel blocker effect on crop muscles

Ion channels are involved in cell volume regulation, cell-to-cell messaging, conduction of action potentials, and muscle contractions. It was hypothesized (Thomson and Holling 1975a) that muscle distortion (i.e., involvement of stretch-activated channels), which is a form of a mechanosensitive channel, is involved in crop lobe muscle contractions and therefore emptying of the crop in *P. regina*.

This hypothesis was substantiated using the peptide toxin from *Grammostola spatulata* spider venom (GxMTx-4), which reduced crop muscle contractions in adult *P. regina* by 58% (Stoffolano et al. 2010). The effect of amiloride at 1 µM produced a similar reduction (58%) of crop contractions, as did the spider peptide toxin. Lane et al. (1991), using patch clamp techniques, showed amiloride blocked a mechanosensitive cation channel in *Xenopus* oocytes. In the present report, the effect of Bztc was tested, as its effect on a membrane channel remains unknown. What ion channels are involved in crop muscle regulation was tested by using specific channel blockers.

Knight (1962) used an unknown physiological saline (presumably with Ca^2+^) while Thomson (1975b) used the saline II and IV of Berridge (1966), both of which contained Ca^2+^. Using *Phormia* saline devoid of Ca^2+^, Liscia et al. (2012) showed crop lobe muscle activity is significantly reduced. The importance of an exogenous source of Ca^2+^ for crop lobe contractions was further substantiated by using the channel blockers amiloride and benzyltrimethylammonium chloride. To our knowledge, destruxins, which constitute a family of cyclic peptides and are produced by various species of entomopathogenic fungi, have not been tested on dipteran visceral muscle. Ruiz-Sanchez et al. (2010), using hindgut and oviduct visceral muscle of *Locusta migratoria*, suggested the effect of destruxins may be dependent for its action on extracellular calcium. Thus, this peptide needs to be tested on the dipteran crop system and may provide information concerning the importance of intracellular versus extracellular Ca^2+^

### Bztc

Based on our results and others, there is little doubt that Bztc acts as a myosuppressin mimic. Lange et al. (1995) first reported Bztc as an agonist of the SchistoFLRFamide (PDVDHVFLRFamide) receptors and demonstrated it inhibited muscular activity of the locust oviducts. Richer et al. (2000) showed that Bztc at 10^-3^ M acted like dromyosuppressin by inhibiting muscle contraction of the adult crop of *P. regina* by 12.6%, while results of our study, using the same concentration, showed an 18.8% decrease. Mispelon et al. (2003) reported Bztc only suppressed heart muscle activity in *D. melanogaster* pupae when it was administered by microinjection, but it produced a response in all three life stages when applied in the absence of hemolymph. Nachman et al. (1996) reported Bztc was a nonpeptide ligand that acted on a peptide receptor. How Bztc acts, and via what mechanism, remains open because the research of Egerod et al. (2003) identified and cloned the two receptors for dromyosuppressin and found that these receptors were not activated by Bztc. If Bztc has an effect on an ion channel, studies using patch clamp techniques should resolve this contradiction. In our study, the IC_50_ value of 10 nM is the minimal amount of Bztc needed to inhibit crop lobe muscle contraction. Furthermore, the IC_50_ value of Bztc is the same as the IC_50_ value of benzyltrimethylammonium chloride, which could potentially illuminate its interaction with receptors on crop muscles leading to inhibition of crop lobe muscle contraction.

### Amiloride

Amiloride blocks T-type Ca^2+^ and Na^+^ epithelial channels. In *Xenopus*, it has been found to block the FMRF-amide activated Na^+^ channel in concentrations as low as 0.6 µM (Lingueglia et al. 1995). In insects, amiloride has been shown to block gustatory responses in several fly species (Liscia et al. 1997; Sadakata et al. 2002; Chen et al. 2010), but our study is the first to show that it significantly blocks supercontractile muscle contraction of the adult crop lobes of a fly and that amiloride-sensitive cation conductances are present on the crop lobe muscles. If the crop continued to contract normally after exposure to amiloride, then calcium channels, and maybe even stretch-activated channels, would not be present. However, the results of our study show application of amiloride significantly decreased the contraction rate and the decrease was in a dose-dependent pattern. These results provide evidence that calcium channels could be present in the crop lobes, possibly reinforcing the hypothesis that stretch-activated channels are also present in these muscles (at least the kind that are permeable to Ca^2+^).

Gadolinium, an ion known to block stretch-activated channels, was not tested due to reports stating gadolinium does not directly block these channels, but instead it may affect the nature of the lipid bilayer of the plasma membrane, indirectly altering the mechano-sensitivity of these channels (Martinac 2004). Reports on false negatives caused by the use of Gd^3+^ have also been reported (Caldwell et al. 1998). They concluded that other anions, like phosphate and carbonate, will bind to free Gd^3+^, other stretch-activated channels are insensitive to Gd^3+^, and Gd^3+^ is not a specific antagonist (Caldwell et al. 1998).

Amiloride was also chosen to measure its IC_50_ value; however, it did not work as efficiently as other compounds tested (e.g. Bztc and benzyltrimethylammonium). Amiloride's IC_50_ value of 0.01 mM was at a higher concentration than the IC_50_ value calculated for both Bztc and benzyltrimethylammonium. The IC_50_ for amiloride in our study of the crop of *Phormia* was 0.01 mM, which is similar to the IC_50_ found in the Chen et al. (2010) study (i.e., 0.02 mM) for inhibition of the gustatory water chemoreceptor response of adult *Drosophila*.

### 9-AC

This substance was used to test for the presence of Cl^-^ channels because it is a reported Cl^-^ channel blocker. As seen in Figure 1, all concentrations tested decreased the contraction rate, and the difference between the rates before and after application were significantly different. Furthermore, although all concentrations significantly reduced crop lobe contraction rate, obtained values did not follow a dose response pattern. It can therefore be assumed that the IC_50_ value is near the lowest concentration tested (0.1 mM). The importance of ClC channels in the larval muscles of *Drosophila* was discussed by Rose et al. (2007), and using 9-AC as the ClC channel blocker, they reported the various effects of these channels on muscle activity and observed the expression of the DmClC-2 gene in larval muscles. Based on the results of our study, the crop supercontractile muscles of *P. regina* may provide another muscle tissue to explore the role of ClC channels in insects.

### Benzyltrimethylammonium chloride

This substance was tested because part of its chemical structure is nearly identical to part of Bztc. When Bztc dissolves in solution, its positively charged amine likely interacts with receptors. In order to test this hypothesis, benzyltrimethylammonium chloride was chosen, as it is nearly identical with a few differences in the amine's R-group. The results showed that Bztc and the benzyltrimethylammonium chloride effects could be superimposed. Thus, they could have a similar, if not the same, mode of action (the amine is essential for the interaction with the receptors and causes a decrease in contraction rate, but the other R-groups are not the essential moieties). This quaternary ammonium compound has a structural resemblance to acetychloline (Abdo 2000) and was targeted by the National Institute of Environmental Health Sciences as a chemical of increasing usage that has potential as an environmental problem (USDH & HS 2000). A search for its use in insects only revealed numerous patents that have been filed with little or no reference to its mode of action in insects. The closest reference found to our study on muscle tissue was on guinea pig ileum muscle, where benzyltrimethylammonium bromide was reported to effectively block muscle contraction normally induced by histamine, potassium chloride, or acetycholine (Strycker and Long 1969). Benzyltrimethylammonium chloride is reported to mimic the action of acetylcholine through its action on muscarinic and nicotinic receptors (Long et al. 1965; Abdo 2000). Our study represents the first report on the effect of benzyltrimethylammonium chloride on insect muscle.

### Controls of crop lobe muscles to date

Knight (1962) noted that the crop was more active when partially filled, and that as the ventriculus emptied, the activity of the crop duct and esophagus also increased. From these observations she deduced that the peristaltic movement that causes food transport is regulated by endogenous mechanisms. Thomson and Holling (1975b) suggested that stretch-sensitive muscle fibers may activate crop movement. Gelperin (1966) showed that the composition of the hemolymph affected crop emptying wrote. The fact that the channel blockers used in our study all significantly decreased crop lobe muscle contractions suggests the following ions play a role in crop function: Cl^-^, type Ca ^2+^, Na^+^, and a FMRF-amide action.

### Concluding remarks

The so-called diverticulated dipteran crop is no simple organ system, and its regulation is proving to be extremely complex. It may not be directly controlled by the nervous/endocrine systems, but surely the supercontratile muscles are modulated by both neuropeptides (Richer et al. 2000; Haselton et al. 2004, 2006) and hemolymph borne factors (e.g., glucose/trehalose levels; Thomson 1975a), serotonin levels (Liscia et al. 2012), and various ions (our study). Most references to this organ system simply refer to it as the crop. Studies like the ours demonstrate the muscles of the crop lobes (P5) are influenced by various chemical factors acting directly on the muscles and presumably act via various membrane channels, and Thomson (1975b) reported a number of sphincter/valves and pumps also involve muscles that need to be further investigated before a comprehensive understanding of both crop filling and emptying is possible. Other results (Liscia et al. 2012) clearly show, using both *in vitro* bioassays and electrophysiological techniques, that an exogenous source of serotonin, acting as a neurohormone, affects the crop lobes differently than that of pump 4.

This study was initially meant to give some insight into how novel insecticides might affect crop filling and emptying, which in turn would impact the fly's ability to properly ingest and digest nutrients. The current use of traditional insecticides is controversial, as most seem to also have negative effects on humans and the environment (Whetstone and Hammock 2007). Chong et al. (2007), using arthropod-selective peptide neurotoxins as selective channel blockers for insect calcium channels, found them to be “high affinity blockers of insect large-conductance calcium activated K^+^(BK_Ca_) channel currents with IC_50_ values of 3–25 nM,” and suggested spider venoms, as are other peptide neurotoxins, may be a novel source of insecticides for biopesticide engineering. The involvement of calcium activated K^+^(BK_Ca_) channels on crop muscles cannot be excluded. Thus, by knowing what ion channels are present in the crop muscle system and what possible peptide neurotoxins could shut down crop activity, it will be possible to better focus research into chemicals/chemical mimics that could inhibit crop contractions causing flies to starve to death without risk to human health and the environment. In addition, the supercontractile muscle system of the adult fly crop now emerges as a novel tissue system to examine the role of not only the different conductances, but also of novel receptor sites.

## Abbreviations:

9-AC,: 9-anthracenecarboxylic acid
Bztc,: benzethonium chloride
IC_50_,: 50% inhibitory concentration
